# The Application of Patient-Derived Xenograft Models in Gynecologic Cancers

**DOI:** 10.7150/jca.46145

**Published:** 2020-07-11

**Authors:** Wenxiao Jiang, Shangdan Xie, Yi Liu, Shuangwei Zou, Xueqiong Zhu

**Affiliations:** Department of Obstetrics and Gynecology, the Second Affiliated Hospital of Wenzhou Medical University, Wenzhou, Zhejiang, 325027, China.

**Keywords:** patient-derived xenograft models, gynecologic cancers, characteristics, application, preclinical utilization

## Abstract

Recently, due to the limitations of cell line models and animal models in the preclinical research with insufficient reflecting the physiological situation of humans, patient-derived xenograft (PDX) models of many cancers have been widely developed because of their better representation of the tumor heterogeneity and tumor microenvironment with retention of the cellular complexity, cytogenetics, and stromal architecture. PDX models now have been identified as a powerful tool for determining cancer characteristics, developing new treatment, and predicting drug efficacy. An increase in attempts to generate PDX models in gynecologic cancers has emerged in recent years to understand tumorigenesis. Hence, this review summarized the generation of PDX models and engraftment success of PDX models in gynecologic cancers. Furthermore, we illustrated the similarity between PDX model and original tumor, and described preclinical utilization of PDX models in gynecologic cancers. It would help supply better personalized therapy for gynecologic cancer patients.

## Introduction

Nowadays, more than 113,520 new gynecologic cancer cases were diagnosed and almost 33,620 mortalities have occurred in the United States 1. Gynecologic cancers include ovarian cancer, cervical cancer, endometrial/uterine cancer, vulvar cancer, and vaginal cancer, according to tumor locations 2. Although treatment strategies have been developed in recent years, the survival rate has not notably improved and many patients still undergo cancer relapse due to the highly heterogeneity in tumors. For example, in recent cancer statistics, five-year relative survival rate for females with ovarian cancer and cervical cancer were reported only about 48% and 66%, respectively 1. Cervical cancer is one of the most common malignancies in females worldwide with a poor overall prognosis for metastatic or recurrent cases 3. Currently, patients with recurrent or metastatic cervical cancer are treated with chemotherapy, but their responses to single and combination therapies still remain poor. Ovarian cancer is a highly malignant cancer and the most deadly cancer in gynecology 4, with almost 75% cases developing relapse or chemoresistance after initial response to platinum-based therapies. Chemo-resistance and recurrence are the most leading causes of death especially in women with high-grade serous ovarian cancer (HGS-OC). Hence, it is urgent to seek for personal and precise therapeutic targets for gynecologic cancers especially ovarian and cervical cancers.

To our knowledge, heterogeneity in gynecologic malignancies contains histopathology, inter-cancer and population heterogeneity, which makes it difficult to obtain a cure with current therapies. Emerging evidence has confirmed the importance of personalized treatment approaches targeting molecular alterations for individual patients. Preclinical researches in gynecologic cancers largely rely on cloned cancer-derived cell lines, cell lines-derived tumor xenografts and animal models. Regretfully, animal models such as mice are so extremely different from humans 5 and insufficiently reflect the physiological situation in humans, subsequently leading to treatment failures in clinical trials 6. Conventional cell lines that are cultured *in vitro* and *in vivo* lose their original tumor characteristics due to genetic and phenotypic alterations when transplanted 7. As a result, patient-derived xenograft (PDX) model has recently been established to overcome these disadvantages, and become the most reliable human cancer model *in vivo* for preclinical research, as it accurately recapitulates molecular, genetic, histological, and chemo-responsive characteristics of original cancer 8-11, improving therapeutic strategies against gynecologic cancers. PDX model has been largely applied to the researches of cancer drug resistance 12 and molecular mechanism of relapsed and metastatic tumors 13,14, assessment of anti-tumor drug efficacy and discovery of new anti-cancer medicines 15.

Currently, more and more evidence has witnessed the application of PDX models in numerous gynecologic cancers including ovarian cancer 16,17, and cervical cancer 18,19, improving our understanding of cancer biology and mechanisms of therapeutic response in gynecologic cancers. Hence, this review is designed to assess the application and current preclinical utilization of PDX models in the field of gynecologic cancer, for providing more opportunities to optimize these models to develop clinical guidelines to manage gynecologic cancer treatment.

## Generation of PDX models

**Figure 1:**
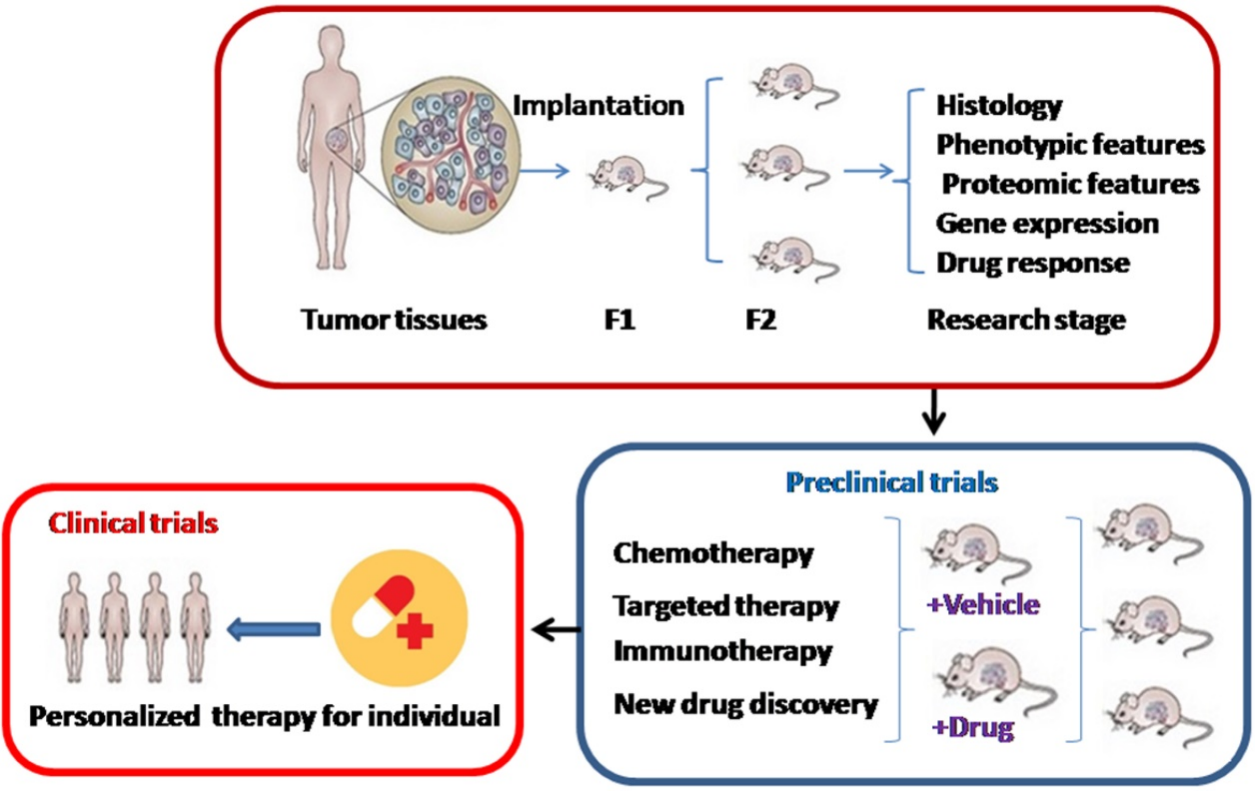
** The development and application of patient-derived xenograft (PDX) models**. F1: Cancer tissues are engrafted directly into immuno-deficient mice. F2: Then cancers are transplanted into a second generation of immuno-deficient mice.

PDX models are acquired by direct engraftment of patient biopsy or surgical dissected tumor tissues into immuno-deficient mice and subsequent transplantation into passage recipient mice (**Figure 1**). Generally, these models are performed through heterotopic or orthotopic implantation. Unlike orthotopic injection, heterotopic implantation occurs when cancer samples are injected into a mouse site independent on the primary cancer location, generally subcutaneously, by sub-renal capsular, in the interescapular region, or through the mammary fat pad 20,21. Most popular models currently offered to patients are subcutaneous-transplantation in immuno-deficient mice, which rarely metastasize and uncommonly simulate the initial tumor microenvironment 22. Subrenal capsule grafting can largely improve tumor engraftment success and reservation of human cancer heterogeneity 23. In contrast, orthotopic-transplant PDX models can generate metastasis and accurately mimic the natural environment of primary tumor, which are usually used for the study of tumor metastasis 24. For most ovarian cancers, research is frequently performed using heterotopically transplanted PDX models, because it is technically easy and can monitor cancer size accurately.

In addition, the most common mouse strains include severe combined immuno-deficient (SCID), non-obese diabetic (NOD)/severe combined immuno-deficient (SCID), NOD/SCID/IL2Rγ null (NSG), and athymic nude mice 25. In gynecologic cancers especially ovarian cancer, NSG and SCID mice are the most frequently used hosts due to their high engraftment rate 26,27. Sometimes nude mice, lacking thymus and T lymphocytes, are also used for gynecologic tumor xenografts because of its cheap expend. Moreover, the time to tumor formation is varied among cancers. For example, the establishment time in high-risk endometrial cancer PDXs was between 2 and 11 weeks 28. It was shorter than the length of tumor establishment in cervical cancer PDX models, whose mean length of time was 32.4 +/- 3.5 weeks and similar to the time in its successive transplantations 18.

Furthermore, it is also important to provide a useful imaging tool for monitoring of PDX cancer models in gynecologic cancers. Apparent diffusion coefficient (ADC) values derived from diffusion-weighted magnetic resonance imaging (DW-MRI) could reflect the cellular environment of biological tissues. In four cervical squamous cell carcinoma PDX models, one group observed that median tumor ADC was negatively related to the fraction of collagen I, suggesting that DW-MRI may be a non-invasive imaging approach for characterizing the stromal microenvironment of cervical cancer 29. Consistently, four cervical cancer PDX models were used to detect the correlation between dynamic contrast-enhanced (DCE) MRI and parameters of the tumor microenvironment, and it was noticed that DCE-MRI provided valuable information on the hypoxic fraction of cervical squamous cell carcinoma 30. In the study of endometrial carcinoma PDX models, Haldorsen et al. 31 described that the positron emission tomography (PET) tracers imaging methods found metastasis at 82% (9/11) of the necropsy mice, suggesting 18F-fluorodeoxyglocose (18F-FDG) is a promising imaging tool for monitoring PDX models in endometrial cancer.

## Engraftment success of PDX models in gynecologic cancers

Engraftment rate is often influenced by multiple factors, including the characteristics of cancer subtypes, host strain, implantation site, primary versus metastatic tumors, patient's treatment status, and preservation of the tumor specimens.

### Cancer subtypes

The engraftment rate is various according to different gynecologic cancers and different tumor types. The engraftment success rate of cervical cancer PDX models was reported from 66.7% to 71.4% 18,19, while the PDXs engraftment rate of endometrial cancer was between 60% and 86% 32,33. As to ovarian cancer PDX models, engraftment success ranged from 45.5% to 100% 34-36. Among uterine sarcomas, a kind of rare and heterogeneous gynecologic tumor, it was identified that the engraftment rate of leiomyosarcomas was 77% (10/13), compared with 29% in carcinosarcomas 37.

The engraftment rate also appears to vary depending on cancer subtypes. For instance, the successful engraftment rate of epithelial ovarian cancer (EOC) PDXs ranged from 45.5% to 48.8% 34,38, which was similar to the engraftment rate of estrogen receptor (ER)-positive relapsed high grade ovarian cancer (HG-OC) PDXs reaching 52.9% 39. Nevertheless, the xenografting success rate of HGS-OC PDX models was up to 83% 40. In a recent study, Wu et al. 16 uncovered that the tumor formation rate in ovarian carcinoma PDX models was 18.52%, among which the tumor formation rate of nonepithelial ovarian tumor PDXs was higher than that of epithelial ovarian tumor (17.39%). Meanwhile, they discovered 100% of tumor formation rate for ovarian germ cell tumor PDX models and 33.33% for metastatic ovarian cancer PDXs. It may be suggested that a more malignancy cancer presents a higher engraftment rate.

### The strain of host

Success rate differs among various host strains. As early as 1993, it was uncovered a more successful engraftment of human tumor PDXs in SCID mice than that in nude mice 41. Recently, a systematic review confirmed that the PDX engraftment success in different mice was nude<SCID<NOD/SCID<NSG, suggesting that more immune compromised mice contribute to a more successful PDX engraftment 42.

Regarding ovarian cancer PDX models, better engraftment efficiency was achieved by implanting cancer samples into SICD mice than into BALB/c nude mice or NSG mice 40. It was noticed that the engraftment rate of ovarian cancer in female CB17 SCID mice was 68% 43, more successful than 45.5-48.8% in nude mice 34,38. Additionally, the engraftment success rate of HGS-OC PDXs with subcutaneous and intra-ovarian bursal implantation into NSG mice was 83% 40, higher than 52.9% in ER-positive relapsed HGS-OC patients with intraperitoneal implantation into female SCID beige mice 39. Consistently, the engraftment success rate of cervical cancer PDX models was identified as 71.4 ±12.5% in NSG mice 18, which was more successful compared with 66.7% in BALB/c nude mice with subrenal capsule implantation 19. However, an additional recent study addressed that animal species, the initiation site of tumor, cancer malignancy degree, cancer stage, cancer type were not related to the tumor formation rate of ovarian carcinoma 16.

### Implantation site

The implantation site is also an important factor affecting engraftment success. The high-risk endometrial cancer engraftment rate in subrenal capsule models was 62.5%, higher than that in subcutaneous models (50%) 28. In EOC PDX models, Colombo et al. 34 implanted cancer tissues into the inter-scapular fat pad of Swiss-nude mice and reported the engraftment success rate was 45.5%, which was slightly lower than 48.8% when EOC specimen was transplanted into subrenal capsule of female BALB/C-nude mice 38.

### Primary versus metastatic tumor

Another important consideration regarding engraftment rate is metastatic tumor. It was noticed that the engraftment success rate in metastatic or recurrent endometrial cancer PDX models was 60% 32, which was lower than 77.8% in high-risk endometrial cancer PDXs including 10 high-grade endometrial cancer models, six serous carcinoma models, one clear cell carcinoma model, and one carcinosarcoma model 28.

### The status of patients

Patients' status also influences the engraftment efficiency. A research delineated that the engraftment success in EOC PDX models was negatively related to the overall survival rate in patients whose tumors were implanted into mice 38, suggesting an inverse correlation between engraftment rate and patients' status. This similar phenomenon was also discovered in other cancers such as lung cancer, showing that high stages (stage III or stage IV), adenocarcinoma and moderately differentiation were related to the PDX engraftment success 44.

### Preservation of the tumor specimens

Considering the effect of the preservation method of tumor specimens to the engraftment success, Alkema et al. 43 created 45 subcutaneous ovarian cancer PDX models from 66 ovarian cancer women in advanced stage (III/IV) and found that the fetal calf serum (FCS)/DMSO-based cryopreservation of ovarian cancer tissues presented a higher take rate of 94% in comparison with vitrification cryopreservation (67%) and using fresh PDX cancer samples (91%), despite of the overall take rate of 68%.

### Other factors

Of note, it was reported that receptor tyrosine kinase-like orphan receptor (ROR1)-positive cells from ovarian cancer PDXs with a high expression of aldehyde dehydrogenase 1 (ALDH1) were more likely to engraft tumor into immune-deficient mice than ROR1-negative ovarian cancer cells, suggesting that ROR1 expression is associated with the engraft success 45. Considering the contribution of mouse pain and stress to engraftment success, ovarian cancer PDX models established orthotopically showed that tumor engraftment rate in analgesia-treated groups was similar to that in control group 26.

## Similarity between PDX model and original tumor in gynecologic cancers

Numerous researches have contributed to the study of concordance between PDX models and primary tumors in phenotypic and proteomic characteristics, gene expression, and drug response.

### Phenotypic and proteomic features

Some papers have confirmed a high resemblance in histology, phenotypic features and molecular markers between gynecologic cancer PDX models and donor tumors including ovarian cancer, cervical cancer, endometrial cancer and leiomyosarcomas 18,19,32,34,37. For instance, established orthotopic HGS-OC PDX models in NSG mice from three donors have been found to maintain their original morphology and molecular marker profile 46. In addition, another research group validated the ability of EOC PDX models to conserve their original phenotypic features and differentiation level, as well as ability to recapitulate the intratumoral heterogeneity of primary cancer 34. Similarly, cervical cancer PDX models have been validated to have highly retention of morphological, histoarchitecture and immunohistochemical features, including similar p16 INK4a and HPV of the original cancers 18.

Proteomic features of these PDX models have been compared with those of original tumors, which is critical for evaluating the therapeutic trial designs and drug response. Using immunohistochemistry, Wu et al. 16 addressed that ovarian cancer PDXs expressed some similar proteins to original cancer such as nervous tissue marker (Syn), epithelial tissue marker (CK7), interstitial tissue marker (Vimentin), tumor protein p53, proliferating cell nuclear antigen, proliferative marker antigen Ki-67, and nuclear factor erythroid 2 like 2 (Nrf2). Earlier evidence also confirmed the positive expression of WT1, PAX8, ER, progesterone receptor (PR), as well as Ki-67 in HGS-OC PDXs similar to those in corresponding tumor 40. It was revealed that desmin and H-caldesmon proteins were expressed in 8 models among 10 leiomyosarcomas PDX models, and the changes in vimentin and cytokeratin expression in carcinosarcomas PDX models varied over the generations 37. Another related evidence revealed that thrombospondin-1 (TSP-1), regulating cell interaction with the microenvironment, was expressed in 10/11 ovarian cancer PDXs and upregulated in the homologous primary tumor, implying similar microenvironment of the PDX and original tumor 47. On the other hand, it is also critical to illustrate the nonhuman component of expression data in PDX models. Liu et al. 48 described several differential protein kinases in ovarian cancer PDXs compared with donor tumor, such as PDGFRA, PDGFRB and CSF1R.

### Gene expression

With the analysis of transcriptome sequencing, one study identified a similarity in gene expression, gene fusion, gene splicing and single nucleotide polymorphisms between ovarian cancer PDX models and their corresponding tumors, and especially observed a consistent rate of 87.19% in gene expression with 19,493 co-expressed genes 16. In the investigation of mRNA differences between nine ovarian cancer PDX models and donor tumors, another research group accounted for 78.4% gene reads in PDX tumor mapping to the human reference genome, higher than 16.3% mapping to the host mice genome, and also identified 1,935 PDX-donor differential genes, which enriched in stroma-specific functions. They further deleted these differential genes and found a stronger transcriptional similarity between ovarian cancer PDXs and original tumors with average correlation coefficient increasing from 0.91 to 0.95 48. Similarly, EOC PDX models were also demonstrated to reserve the copy number change characteristics of the primary cancer over several passages and maintain an oligoclonal structure, implying that ovarian cancer PDX models reserve at least part of the clonal diversity of the original tumor 34. In cervical cancer, it was demonstrated that all PDX tumors including serially passage models maintained the genomic DNA alterations observed in original cancers 19. These suggest that PDX models of gynecologic cancers displayed a high concordance with original tumors in gene expression and the differential expressed genes were mainly related to stroma functions.

In analyzing the gene mutation of ovarian cancer, one research group established HGS-OC PDXs and reported a similar copy number variation to original tumor, and that the frequency of TP53 alterations in PDXs was 93%, BRCA1 mutation was 13%, BRCA2 mutation was 8%, consistent with the analysis in The Cancer Genome Atlas (TCGA) data 49. Similarly, another study indicated that the rate of TP53 mutations in ten HGS-OC PDXs was 100%, BRCA1 mutation with methylation was 20%, and BRCA2 mutation was 30% 40. A good resemblance in genetic mutation profiles and gene expression was also demonstrated in high-grade endometrial cancer PDX models compared with primary tumors 28. While, using whole-genome low-coverage sequencing, another group documented that the similarity in copy number profiles between leiomyosarcomas PDX models and donor tumors ranged from 57.7% to 98.2% and between carcinosarcomas PDX models and donor tumors was between 47.4% and 65.8% 37.

### Drug response

Considering chemotherapy response, a highly concordance of cisplatin and carboplatin sensitivity between HGS-OC PDX models and donor patients was detected 40,49. In one study, Oh et al. 19 established 14 cervical cancer PDX models and confirmed a high expression of human epidermal growth factor receptor-2 (HER2) in serially passaged PDX models similar to that in original cancer, implying that PDX models can be used to study the response to HER2 target therapy in cervical cancer patients.

## Preclinical utilization of PDX models in gynecologic cancers

PDX models have been used to explore tumor mechanism, identify candidate drugs and monitor therapeutic response in gynecologic cancers for decades.

### Standard chemotherapeutics in PDX models of gynecologic cancers

Drug resistance is one of the major contributing factors that determine the efficacy of chemotherapy in patients. In order to determine gynecologic cancer sensitivity or resistance to standard chemotherapy, an increasing number of researches on PDXs have been carried out. In order to identify the chemo-resistant patients in ovarian cancer, Dobbin et al. 50 compared six pairs of ovarian cancer PDXs which were divided into two groups of combined carboplatin/paclitaxel treatment and no treatment, showing a consistent disparity in genetic profile after therapy, suggesting that heterogeneity of PDX models can be used to identify the chemo-resistant population in ovarian cancer. Similarly, by creating HGS-OC PDX models, paclitaxel/carboplatin standard chemotherapy was demonstrated to markedly reduce PDX tumor weight in comparison with the phosphate buffered saline group with statistical significance 38. Furthermore, a study in ovarian cancer PDX models validated that combination of ifosfamide and paclitaxel was more favorable in inhibiting tumor growth than the treatment of combined carboplatin and paclitaxel 51. Interestingly, in order to overcome chemo-resistance to paclitaxel, Byeon et al. 52 developed a hyaluronic acid-labeled poly (d,l-lactide-co-glycolide) nanoparticle (HA-PLGA-NP) incorporating paclitaxel and focal adhesion kinase (FAK) siRNA and demonstrated that this compound dramatically suppressed cancer growth and overcame chemo-resistance in comparison with paclitaxel alone in a drug-resistant EOC PDX model. Exportin-1 (XPO1) is a nuclear exporter, which mediates nuclear export of various cancer inhibitors. Another study developed platinum-resistant ovarian cancer PDX models and revealed that a XPO1 inhibitor selinexor significantly reduced model tumor growth and benefited mice survival no matter monotherapy or in combination with platinum 53. Furthermore, it has been known that pregnancy-associated plasma protein-A (PAPP-A) suppression is associated with platinum sensitivity in chemotherapy. Torres et al. 54 developed ovarian cancer PDXs in female SCID/beige mice, which were divided into two cohorts: high PAPP-A (n = 5) group and low PAPP-A (n = 2) group, and then injected these models with saline, carboplatin/paclitaxel + anti- PAPP-A monoclonal antibody inhibitor (mAb-PA), or carboplatin/paclitaxel + IgG2a (control antibody) in randomization. They then examined these models with ultrasound after 28 days of treatment and found that carboplatin/paclitaxel combined with mAb-PA induced tumor degeneration below baseline in one high PAPP-A PDX model and inhibited tumor growth in another three models compared with carboplatin/paclitaxel + IgG2a, while no low PAPP-A PDX models regressed tumor below baseline, suggesting that mAb-PA sensitized ovarian cancer to carboplatin/paclitaxel chemotherapy. In line with this, Garrett et al. 55 established HGS-OC PDX models by transplanting tumor tissues into 6-week old female NOD/SCID mice, which then treated with carboplatin/paclitaxel for 21 days, and found that LBH589, a novel histone deacetylase inhibitor panobinostat, decreased the growth of ovarian cancer in PDXs and promoted the effect of carboplatin/paclitaxel therapy in one of three PDX models. Another group developed 14 clinically annotated and molecularly characterized luciferized ascites-derived ovarian cancer PDX models, and demonstrated the consistency of response to carboplatin and paclitaxel evaluation across different assay platforms, including bioluminescent imaging (BLI) and plasma CA125 levels or LINE-1 biomarkers, suggesting that BLI can be as a platform for proof-of-concept efficacy and biomarker studies and for validation of novel therapeutic strategies in ovarian cancer 56.

Another utility of PDX models for standard chemotherapy is to explore biomarkers or pathways related to chemo-resistance that may help understand the chemoresistance mechanisms. One group established 42 ovarian cancer PDX models with different sensitivity to cisplatin, revealing that cyclin dependent kinase 12 (CDK12) mRNA expression was negatively related to cisplatin sensitivity and positively associated with tumor recurrence rate in high-grade serous/endometrioid ovarian cancer PDX samples *in vivo*, suggesting that the CDK12 may be an important gene in ovarian cancer cell resistance to cisplatin 57. Moreover, Wnt/β-catenin signaling pathway was demonstrated to be associated with platinum resistance of HGS-OC PDX models, with retention of stem-like properties, implying that Wnt/β-catenin inhibitor iCG-001 induced cisplatin chemosensitivity of ovarian cancer cells 58. In addition, Li et al. 59 established 3 chemoresistance and 4 chemosensitivity HGS-OC PDX tumor models by implanting cancer tissues subcutaneously into mice which were then treated with paclitaxel and carboplatin. In these models, they identified 146 up-regulated genes and 54 down-regulated genes in chemoresistance group, including genes SAP25, HLA-DPA1, AKT3, and PIK3R5 by RNA sequencing analysis, and also found 39 mutation sites only shown in chemoresistance group, including important gene mutation of TMEM205 and POLR2A by whole exome sequencing analysis, suggesting these differently expressed genes and mutations could be provided to predict chemotherapy response.

### Targeted therapy of PDX models in gynecologic cancers

Accumulating evidence has demonstrated that PDXs play a vital role in the trial of targeted therapeutic drugs, which helps provide individual treatment for gynecologic cancer patients.

#### PARP inhibitors

Up to date, although polyadenosine diphosphate ribose polymerase (PARP) inhibitors (PARPi) have been applied for clinical therapy for many years, the therapeutic resistance to them especially in ovarian cancer treatment is still a clinical problem. Herein, more and more researchers turn the PARPi study to the PDX models. Nowadays, FDA-approved PARP inhibitors for treating HGS-OC include olaparib, rucaparib and niraparib 60. Almost half of the EOCs exhibit defective DNA repair via homologous recombination containing BRCA1/2 (BRCA1 and BRCA2) mutations and formation of Rad51 foci after DNA damage. Homologous recombination deficiency is an important target for PARPi to treat ovarian cancer 61. To date, an increasing number of researchers have utilized PDX models to study the biomarkers related to the resistance to PARPi in ovarian cancers. For example, it was found that methylation of all BRCA1 copies was correlated with the response to the PARPi rucaparib in 12 BRCA1-methylated HGS-OC models 62. Shah et al. 63 examined 3 ovarian cancer PDX models by implanting fresh omental ovarian tumor nodules into SCID mice and showed that the PDXs' response to PARPi was related to the ex vivo ionizing radiation (IR) assay. Notably, an increased expression of euchromatic histone-lysine-N-methyltransferases 1 and 2 (EHMT1/2, akaGLP/G9A) has been validated in PARPi-resistant HGS-OC PDX models, implying its association with the resistance to PARPi of HGS-OC 64.

Parmar et al. 65 utilized 14 characterized luciferized HGS-OC PDX models and revealed that 13 models were resistant to olaparib monotherapy, among which 4 models presenting BRCA1 mutation, and olaparib in combination with a checkpoint kinase 1 (CHK1) inhibitor prexasertib contributed to tumor inhibition in olaparib-resistant models. Consistently, it was identified that combined use of PARPi and ATR/CHK1 inhibitor exhibited a more effective antitumor activity than PARPi monotherapy in the established recurrent BRCA-mutant (BRCAMUT) HGS-OC PDX models 66. Whilst, olaparib and chloroquine (CQ) were shown to induce synergistic antitumor activity and promoted drug resistance via autophagy in ovarian cancer PDX models 67. In contrast, AlHilli et al. 68 developed five homologous recombination deficiency HGS-OC PDX models intraperitoneally in 35 female SCID mice to evaluate the antitumor role of niraparib, and found that niraparib monotherapy in one of two PDX models with deficient BRCA2 and in one PDX with Rad51C promoter methylation induced cancer regression, although these models were failed to promote response to carboplatin/paclitaxel chemotherapy.

#### VEGF inhibitor

Bevacizumab is a monoclonal antibody against vascular endothelial growth factor A (VEGFA), and has been widely used in cancer second line therapy as an anti-angiogenic drug. A research using three cisplatin-relapsing ovarian cancer PDX models based on the presence of activation of the RAS/RAF/MEK/ERK axis and PI3K pathway, p53 mutation and lack of phosphatase and tensin homolog (PTEN) expression, confirmed that triple combination of bevacizumab, MEK162 (a MEK inhibitor) and paclitaxel displayed a more effective antitumor activity than any double drug combination for relapsing ovarian tumors in second line treatment 69. Meanwhile, another study used 11 EOC PDXs by transplanting tumor tissues orthotopically in the peritoneal cavity of nude mice to evaluate the activity of cediranib (a pan-VEGFR RTK inhibitor) alone or combined with chemotherapy, showing that different EOC PDX models displayed dissimilar response to cediranib, and that combination of cediranib and cisplatin increased the model mice survival and inhibited ascites and metastases while cediranib alone just exerted the decreasing effect on ascites but not on tumor dissemination in advanced EOC PDX 70.

#### PI3K/Akt/mTOR inhibitor

Several inhibitors of PI3K/Akt/mTOR pathway have been clinically applied to some malignancies including ovarian cancer, and c-Met receptor has been found to promote PI3K/Akt/mTOR pathway and to play an important role in drug resistance 71. Activation of PI3K/Akt/mTOR pathway has been identified to be involved in chemotherapy resistance or anti-EGFR/HER2 therapies, and PTEN inhibition activates the PI3K/Akt/mTOR pathway. In a low-grade serous ovarian cancer (LGSOC) PDX model with lack of PTEN expression, two PI3K/Akt/mTOR inhibitors (PF-04691502 and PF-05212384) added to cisplatin or paclitaxel promoted the activity of chemotherapy alone in LGSOC models, suggesting that PI3K-mTOR inhibitors contributed to the chemotherapy sensitivity to cisplatin or paclitaxel 72. An *in vivo* study in one ovarian cancer PDX model revealed that the combination of crizotinib (a c-Met inhibitor) and gedatolisib (a dual PI3K-mTOR inhibitor) exerted a more superior antitumor activity than single agent, while crizotinib alone presented no antitumor activity, and gedatolisib alone only displayed some marginal activity, suggesting that crizotinib promoted the activity of gedatolisib in treating ovarian cancer 73. Another research using a large cohort of human uterine sarcoma samples (288) and identified the most promising target phospho-S6 ribosomal protein (p-S6) among 5 common druggable targets. In order to investigate this target, they developed 5 leiomyosarcoma PDX models and uncovered that PI3K/mTOR inhibitors (BEZ235, also known as dactolisib) therapy inhibited cancer growth of 4/5 leiomyosarcoma PDX models, and the 4 responding models presented basal p-S6 expression but nonresponding model showed negative score, suggesting that dual PI3K/mTOR inhibition is a promising therapeutic strategy in uterine leiomyosarcoma, and p-S6 expression can be used for predicting its response 74.

#### RTK inhibitor

Receptor tyrosine kinases (RTKs) are a subclass of tyrosine kinases that catalyse the transfer of a phosphate from ATP to a hydroxyl group of a tyrosine residue. Nowadays, 58 RTKs have been identified in human beings, and classified to 20 subfamilies, such as receptors of ERBB, insulin, platelet derived growth factor (PDGF) and vascular endothelial growth factor (VEGF), playing an important role in cancer progression 75,76. Therefore, RTKs inhibitors are frequently used to treat tumors. Ponatinib is a small molecule multi-tyrosine kinase inhibitor clinically approved for anticancer therapy. One research group established a rare malignant ovarian cancer small cell carcinoma of the ovary hypercalcemic type (SCCOHT) PDX model and demonstrated that ponatinib delayed tumor doubling time and decreased final tumor volume in this PDX model by 58.6% and 42.5%, respectively 77. In addition, erlotinib is also a RTK inhibitor, which inhibited the activation of epidermal growth factor receptor (EGFR), and has been administrated to treat many cancers. It exerted its antitumor function by inhibiting angiogenesis and consequently impairing intratumoral microcirculation 78. Another research group established an EGFR-overexpressing clear cell ovarian carcinoma PDX, and indicated that erlotinib markedly reduced tumor weight in this model 38.

#### HER2 inhibitor

HER2 belongs to the epidermal growth factor (ErbB, HER) family and is encoded by proto-oncogene ERBB2 on chromosome 17 79. Activated HER2 can promote cancer cell growth and survival and induce reprogramming of tumor metabolism. The HER2 receptor is overexpressed in various cancers, being an attractive target in cancer therapy. Trastuzumab and lapatinib, as US Federal Drug Administration-approved HER2 inhibitors, are commonly used to administrate HER2-overexpressing breast cancers. In 14 established HER2-amplified cervical cancer PDX models, combined treatment of trastuzumab and lapatinib reduced 50% of PDX tumor weight compared with the untreated control, identifying that trastuzumab and lapatinib suppressed cancer growth in the HER2-overexpressed PDXs 19. A recent study transplanted fresh specimens from naïve-treatment ovarian cancer females intraperitoneally into female SCID beige mice to establish 3 ovarian cancer PDX models and then grouped them into untreated, pertuzumab treated, and carboplatin/paclitaxel treated groups, discovering that pertuzumab inhibited tumor growth but did not induce tumor regression, and carboplatin/paclitaxel chemotherapy decreased about 25% of tumor volume, compared with untreated group increasing tumor volume with 4-4.5 fold over the 4 weeks. Meanwhile, the co-administration of HER2-targeted therapy and carboplatin/paclitaxel chemotherapy contributed to a significant tumor regression after 6 weeks in comparison with single chemotherapy, suggesting that HER2-targeted therapy sensitized ovarian cancer response to chemotherapy of carboplatin and paclitaxel 27.

For observing the anti-metastatic function of HER2-targeted therapy, it is important to develop orthotopic PDX models since metastatic and primary tumors in an orthotopic model may exert differential chemosensitivity 80,81. For instance, a benzamide histone deactylase inhibitor entinostat displayed no tumor inhibition in HER-2 expressing cervical carcinoma PDX model in a subcutaneous nude mouse, and also did not suppress original tumor growth in the orthotopic PDX model. But, entinostat alone notably inhibited the metastatic tumor burden in comparison with the control 80.

#### Other target therapy

Gemcitabine is an analog of deoxycytidine, and has been widely used for anticancer treatment. Trabectedin is a cytotoxic agent derived from Caribbean sea squirt with the antitumor activity in treating several cancers including ovarian cancer 82. Pegylated liposomal doxorubicin (PLD) is a formulation of doxorubicin encapsulated in liposomes as an anthracycline topoisomerase inhibitor and has been commonly used for the treatment of many solid malignancies, including recurrent or progressive gynecologic cancers 83. Erriquezet al. 84 implanted pairs of HGS-OC samples from the same patients before and after platinum-based neo-adjuvant chemotherapy into immunocompromised mice, and then treated these xenograft mice equally with carboplatin, gemcitabine, PLD and trabectedin, showing that naïve HSG-OC PDX models displayed response to carboplatin, trabectedin, gemcitabine and PLD, while carboplatin treated PDXs propagated from a tumor mass of the same patient, lost response to trabectedin, gemcitabine and PLD. It suggested that ovarian cancer having been treated with chemotherapeuticc drug may presents chemo-resistance to second line chemotherapy.

### Immunotherapy response of PDX models in gynecologic cancers

With the emerging of tumor immunotherapies and vaccines for tumor therapy, PDX models potently evaluating the preclinical activity of these immunotherapies are more and more important. However, during the development of these PDX models for studying immunotherapies, the unintended formation of human lymphoma is being a potential problem. Butler et al. 85 established ovarian cancer PDX models by injecting species from 568 ovarian cancer patients intraperitoneally into SCID mice and indicated that rituximab, an anti-CD20 antibody, decreased CD45-positive cells incidence in subsequent PDX lines from 86.3% (no rituximab) to 5.6% (rituximab), and reduced lymphoma rate from 11.1% to 1.88%.

Chimeric antigen receptor (CAR) -T cells treatment may be ineffective for solid tumors, since vascular barriers hamper CAR-T cells from reaching the tumor site. Deng et al. 86 used subcutaneous ovarian cancer mouse xenograft models and ovarian cancer PDX models, and reported that combretastatin A-4 phosphate (CA4P), a vascular disrupting agent (VDA), significantly enhanced the therapeutic efficiency of the CAR-T cells, providing a new potential strategy for CAR-T cells treatment in ovarian cancer.

Using RNASeq analyses, another study identified the upregulation of antigen presenting pathways in both ovarian cancer PDXs and original tumors, hinting a strong functional conservation between them. Among the tested 30 neoantigens, they discovered that a core of six neoantigens defining a potent autologous T cell activation inhibited cancer growth 87. Similarly, recent study used orthotopically transplanted HGS-OC tissues and matched autologous-expanded tumor infiltrating lymphocytes (TILs) into NSG mice to create humanized TIL/PDX models for evaluating the anti-tumor effect of immunomodulating therapies against autologous-tumors. In this study, the mice were accepted with TIL infusion alone, TIL infusion + anti programmed cell death protein 1 (PD-1), or vehicle, and mice treated with TILs and anti-PD-1 decreased tumor volume and increased overall survival 46. Another group utilized ovarian cancer PDX models and found a promising therapeutic target Ephrin-A4 (EFNA4) with the identification of E-cadherin (CD324) as a surface antigen able to enrich TIC, and revealed that an antibody-drug conjugate (ADC) containing anti-EFNA4 monoclonal antibody conjugated to the DNA-damaging agent calicheamicin inhibited tumor growth 88. The results of these studies suggest that PDX models of gynecologic cancers especially ovarian cancers play important roles in evaluating human response to immunotherapy treatment and designing optimal clinical trials.

### Other therapies of PDX models in gynecologic cancers

CX-5461 is an RNA polymerase I (Pol I) inhibitor, which exerts its action by inhibiting ribosomal DNA transcription. For studying its antitumor activity in ovarian cancer, one group established 5 ovarian cancer PDXs from 5 advanced papillary serous ovarian cancer patients and uncovered that these models displayed differential response to CX-5461 treatment, with complete response in 1 model, 55% reduction in cancer volume in 1 model, stable disease in 1 model and tumor growing in 2 models after 45 days 89, suggesting that more PDX models are need to carry out, and potential biomarkers are required to find in the study of ovarian cancer response to CX-5461. CUB-domain containing protein 1 (CDCP1) is a cell-surface protein and has been identified to be overexpressed in multiple tumors including clear cell ovarian cancer. It was noticed that in 3 established HGS-OC PDXs, the antibody to CDCP1 dramatically inhibited cancer growth in these ovarian cancer PDX models 90. Furthermore, sphingosine kinase 1 (SK1) inhibitor, FTY720, has been indicated to dramatically attenuate tumor weight in ovarian cancer cell lines (A2780 and SKOV3ip1) xenograft models and a clear cell ovarian carcinoma (CCC) PDX model 91, as well as in cervical cancer PDX models 92, supporting FTY720 as a potential therapeutic agent for gynecologic cancers. Furthermore, two EOC PDX models were used to confirm the activity of itraconazole in ovarian cancers and revealed that combined treatment of itraconazole and paclitaxel markedly attenuated cancer weight, decreased microvessel density of PDX tumor as well as suppressed hedgehog and mTOR pathways in comparison with the control, paclitaxel-alone, or itraconazole-alone groups, suggesting that itraconazole suppressed endothelial cells rather than cancer cells by targeting several signaling pathways including angiogenesis, hedgehog and mTOR pathways 93.

### PDX models for discovering new therapeutic drugs in gynecologic cancers

Another utility of PDX models in gynecologic cancers is to discover novel therapeutic drugs, with greater predictive value for incipient drug testing. In 2015, considering the activity of Mullerian inhibiting substance (MIS) in suppressing the growth of stem-like ovarian cancer cells, one research group devised peptide modifications to human MIS (LRMIS) and delivered it with adeno-associated virus (AAV) to validate its anti-tumor function in chemoresistant serous ovarian adenocarcinoma PDX models from ascites, showing that AAV9-LRMIS monotherapy notably reduced cancer growth without signs of toxicity in 3/5 these PDXs, hinting AAV9-LRMIS as an effective agent for chemoresistant serous ovarian cancer patients 94. Cell division cycle 25B (CDC25B) has been identified to be correlated with poor prognosis of ovarian cancer. BAY-876, as a new-generation inhibitor of glucose transporter 1 (GLUT1), is overexpressed in ovarian cancer but has not been assessed in preclinical animal models. With the use of ovarian cancer PDXs, CDC25B inhibitor WG-391D 95, and BAY-876 96 have been proved to regress ovarian cancer growth in PDXs.

### PDX models for studying mechanism of gynecologic cancer

PDX models also exhibit a critical role in researching cancer mechanism and biomarkers related to cancer development. SET and MYND domain-containing protein 3 (SMYD3), a histone methyltransferase, is a promising epigenetic therapeutic target and has been found to be upregulated in a variety of human cancers. Using ovarian cancer PDX models, SMYD3 was identified to induce ovarian cancer growth 97, and to promote metastasis and reduce ascites volume in ovarian cancer PDX models 98, suggesting that SMYD3 may be related to the development of ovarian cancer. Additionally, an *in vivo* research using cell-line xenograft and EOC PDX models revealed that cyclin-dependent kinase 7 (CDK7) increased ovarian cancer weight via regulation of cell proliferation and apoptosis 99. Of note, Erriquezet al. 100 utilized ovarian cancer PDX models and found an overexpression of topoisomerase II alpha (TOP2A) in cells from xenograft after the treatment of pegylated liposomal doxorubicin (PLD), suggesting that TOP2A may be involved in the pathogenesis of ovarian cancer response to PLD.

## Conclusions

Current evidence has demonstrated that gynecologic cancer PDX model is a useful tool for predicting response to chemotherapy, targeted therapy, immunotherapy, as well as discovering new drugs, for better designing optimal clinical trials, because of its high concordance with original tumor in phenotypic and proteomic characteristics, gene expression, and drug response. However, when it is hoped to provide personalized and precise therapy to each individual cancer patient using PDX models, some limitations and challenges must be mentioned and overcome, which include variable engraftment rates, enormous time and resources taken for PDX generation, high cost, loss of immune systems, and uncertainty of the effect of tumor microenvironment on drug efficacy. Furthermore, except for ovarian cancer PDXs, uterine cancer and other gynecologic cancer PDX models are so few that they cannot benefit these patients with personalized treatment regime. Herein, international collaborative networks should work together to overcome these drawbacks for making the real personalized treatment to come true.

## Abbreviations

EOC: epithelial ovarian cancer
HGS-OC: high-grade serous ovarian carcinoma
HG-OC: high grade ovarian cancer
ER: estrogen receptor
PR: progesterone receptor
TSP-1: thrombospondin-1
ROR1: receptor tyrosine kinase-like orphan receptor
SCID: severe combined immuno-deficient
NOD: non-obese diabetic
TCGA: The Cancer Genome Atlas
